# Cymbopogon citratus showing nematicidal activity against Heligmosomoides polygyrus bakeri

**DOI:** 10.1590/S1984-29612024079

**Published:** 2024-12-20

**Authors:** Viviane Souza Campos, Letícia Oliveira da Rocha, Hassan Jerdy Leandro, Teresa Pontes, Fábio Conceição de Oliveira, Eulógio Carlos Queiroz de Carvalho, Leonardo Siqueira Glória, Clóvis de Paula Santos

**Affiliations:** 1 Laboratório de Biologia Celular e Tecidual, Centro de Biociências e Biotecnologia, Universidade Estadual do Norte Fluminense Darcy Ribeiro – UENF, Campos dos Goytacazes, RJ, Brasil; 2 Laboratório de Patologia Veterinária, Centro de Ciências Tecnológica e Agropecuárias, Universidade Estadual do Norte Fluminense – UENF, Campos dos Goytacazes, RJ, Brasil; 3 Laboratório de Microscopia, Universidade Federal do Sul e Sudoeste do Pará – UNIFESSPA, Xinguara, PA, Brasil; 4 PIC Pig Improvement Company – Genus plc, Hendersonville, TN, United States

**Keywords:** Cymbopogon citratus, Heligmosomoides polygyrus bakeri, gastrointestinal nematodes, anthelmintic, anti-inflammatory, Cymbopogon citratus, Heligmosomoides polygyrus bakeri, nematoides gastrintestinais, anti-helmíntico, anti-inflamatório

## Abstract

This paper describes a novel *in vivo* study of *Cymbopogon citratus* (lemon grass) to assess its anthelmintic activity. To this end, C57BL/6 mice were separated into three groups: G1: uninfected; G2: negative control infected with *Heligmosomoides polygyrus bakeri* and administered with 3% dimethyl sulfoxide (DMSO); and G3: infected with *H. polygyrus bakeri* and treated with *C. citratus* aqueous extract (50mg/kg). The extract and *H. polygyrus bakeri* were administered via gavage and the anatomo-histopathological evaluation of the animals took place after necropsy and organ removal. In addition, the number of eggs per gram of feces (epg) and of adult parasites in the small intestine of each animal, as well as blood cell counts, were assessed. The *in vivo* assay revealed a reduction in the epg (54%), number of adult nematodes (89%), number of eosinophils, and intestinal lesions in mice treated with *C. citratus*. These results suggest that the crude aqueous extract of *C. citratus* at the dose evaluated here has anthelmintic and possibly anti-inflammatory properties, given its effectiveness against gastrointestinal *H. polygyrus bakeri* nematodes and the recovery of damaged tissues. Therefore, this plant shows potential to control gastrointestinal nematodes.

## Resumo

Este artigo descreve um novo estudo *in vivo* de *Cymbopogon citratus* (capim limão) para avaliar sua atividade anti-helmíntica. Para tanto, camundongos C57BL/6 foram separados em três grupos: G1: não infectados; G2: controle negativo infectado com *Heligmosomoides polygyrus bakeri* e administrado com 3% dimetilsulfóxido (DMSO); e G3: infectados com *H. polygyrus bakeri* e tratados com extrato aquoso de *C. citratus* (50mg/kg). O extrato e *H. polygyrus bakeri* foi administrado por gavagem, e a avaliação anatomo-histopatológica dos animais ocorreu após necropsia e retirada dos órgãos. Além disso, foram avaliados o número de ovos por grama de fezes (opg) e de parasitas adultos no intestino delgado de cada animal, bem como a contagem de células sanguíneas. O ensaio *in vivo* revelou redução no OPG (54%), número de nematoides adultos (89%), número de eosinófilos e lesões intestinais em camundongos tratados com *C. citratus*. Estes resultados sugerem que o extrato aquoso bruto de *C. citratus* na dose avaliada possui propriedades anti-helmínticas e anti-inflamatórias, dada a sua eficácia contra nematoides gastrointestinais de *H. polygyrus bakeri* e na recuperação de tecidos danificados. Portanto, essa planta apresenta potencial para controlar nematoides gastrintestinais.

## Introduction

The study of medicinal plants provides opportunities for the development of alternative ways to control parasites in livestock and counteract the increasing incidence of anthelmintic resistance. Studies have shown the *in vitro* effects of secondary molecules obtained from different plant species (Elandalousi et al., 2013; Bachaya et al., 2009; Oliveira et al., 2009; Wabo Poné et al., 2010; Hussain et al., 2011; Kanojiya et al., 2015; Palacios-Landín et al., 2015). In addition to analyzing the *in vitro* effects, the activity of these molecules on specific nematode species that parasitize several botanical species has also been examined through *in vivo* assays (Hussain et al., 2011; Ahmed et al., 2013; Wabo Poné et al., 2009; Vatta et al., 2011; Palacios-Landín et al., 2015). Thus, analyses are required to examine the parasite-host relationship after treatment with botanical extracts.

The validation of new molecules for the control of nematode infection *in vivo* is conducted through assays with host-specific parasite species, and *Heligmosomoides polygyrus bakeri* is one of the models most widely used in such assays (Robinson et al., 1989; Behnke et al., 2009; Asojo et al., 2018). The similarity and taxonomic proximity with parasitic nematodes of ruminants make this helminth a target of *in vitro* tests for validation of treatment with medicinal plants, as well as in preclinical tests (Stepek et al., 2005; Githiori et al., 2006).

*Cymbopogon citratus* (lemon grass) is a herbaceous plant (Poaceae) native to tropical regions of Asia, mainly India (Gupta & Jam, 1978). This plant has several pharmacological properties, including anti-amoebic, anti-bacterial, anti-diarrheal, anti-filarial (anthelmintic), anti-fungal, and anti-inflammatory (Shah et al., 2011). With regard to its anthelmintic properties, studies have shown activity against helminths in dogs (Ritter et al., 2012), plant nematodes (Oka et al., 2000; Barbosa et al., 2010; Fabiyi et al., 2018; Gonçalves et al., 2022), and nematodes in the gastrointestinal tracts of ruminants (Almeida et al., 2003; Silva et al., 2005; Macedo et al., 2015, 2019; Rocha et al., 2020, Aderibigbe & Idowu 2020).

In terms of anti-inflammatory properties, several researchers have identified an association with polyphenols (Costa et al., 2016) and with phytosterol-type steroids (García et al., 1999; Aldini et al., 2014; Yuan et al., 2019). However, to date, no studies have simultaneously evaluated the anthelmintic and anti-inflammatory properties of *C. citratus*. This analysis focused on anthelmintic activity, aiming to contribute to the existing body of knowledge.

## Materials and Methods

### Collection and identification of botanical material

*Cymbopogon citratus* (H.8225) was collected from the greenhouse at the Center for Biosciences and Biotechnology – CBB, State University of Northern Rio de Janeiro – UENF, in Campos dos Goytacazes, state of Rio de Janeiro, Brazil. A voucher specimen (H8225) was deposited in the UENF herbarium, and plant names were verified using The Plant List (2024).

### Experimental mice and preparation of crude botanical extract

To obtain the aqueous extract of *C. citratus*, 1 kg of the plant’s leaves were collected, crushed, and oven-dried at 30 °C for 72 hours. After drying, 6L of distilled water was added and the sample was macerated for 48 hours. The extract was then filtered, frozen, and lyophilized to obtain a dry mass, which was stored in a Falcon tube at -20 °C (Rocha et al., 2020). The dry mass obtained was two grams. The concentrations used in the treatments with crude extracts were obtained from 1g/mL stock solutions of the *C. citratus* aqueous extract.

Female mice (n=21) of the C57BL/6 inbred strain, four weeks old, weighing between 20 and 25g, were provided by the university’s central animal breeding facility. The animal experiment protocols were reviewed and approved by the institution’s Ethics Committee on Animal Use to ensure proper ethical and scientific procedures (Approval Number: 211).

### Anthelmintic assay in mice

Testing was carried out at the animal experimentation facility in Building P2 of UENF. The animals were divided into three groups, each with a total of seven mice: uninfected mice (G1); control mice infected and treated with 3% dimethyl sulfoxide (DMSO) (G2); and mice infected and treated with crude aqueous extract of *C. citratus* (G3).

Each infected mouse received 200 infective larvae of *H. polygyrus bakeri* in a volume of 0.2 mL of distilled water via gavage. Mating and egg-laying occur near the tenth day after infection (Johnston et al., 2015). Therefore, feces were collected starting on day nine post-infection to confirm infection. On the tenth day after the administration of L3 egg-laying occurred, and the extract was administered the next day. The doses of *C. citratus* aqueous extract were administered via gavage according to the weight of each animal and the extracts were diluted in 3% DMSO.

Two doses of the aqueous extract were administered at a concentration of 50mg/kg on days zero and five after confirming *H. polygyrus* infection. The dose was selected based on data reported for *C. citratus in vitro* study that this dose would provide biological activity against gastrointestinal nematodes in sheep (Rocha et al., 2020). A higher dose tested *in vitro* was used for *in vivo* testing. To evaluate the epg count, fecal samples were collected on the day *C. citratus* aqueous extract was administered, and on days five, seven, ten, and 15 thereafter. Two grams of feces were used for epg, according to the modified technique of Gordon & Whitlock (1939). On day 15, the animals were euthanized in a CO_2_ chamber, followed by necropsy to inspect their organs.

Adult nematodes were collected and quantified after inspection of the small intestine. Blood samples were collected to assess hematocrit and white blood cell count and assess the influence of treatment on different blood parameters.

### Anatomo-histopathological analysis

The anatomo-histopathological analysis was conducted using the paraffin embedding technique to verify the mucosal lesions after necropsy. After collecting the organs (kidney, liver and intestine), the samples were fixed in neutral buffered formalin at 10%, trimmed using a scalpel to enable them to fit into an appropriately labeled tissue cassette, followed by dehydration in different alcohol concentrations, clearing in xylene, and paraffin infiltration. Samples were then sectioned (5µm), stained with hematoxylin-eosin and mounted. Slides were assessed using a Nikon Eclipse 80i camera coupled to an optical microscope, and *NIS Elements* software.

### Statistical analysis

The results were subjected to a one-way analysis of variance (ANOVA). Means and standard error were determined with the aid of the GraphPad Prism 5.0 program, and mean values were compared using Tukey’s test at a significance level of p<0.05. For the anthelmintic assay, treatment efficacy was determined using the following formula: FECR% = 100 X (1– Xt2/ Xt1 Xc1/ Xc2) according to Dash et al. (1988), where Xt and Xc represents the arithmetic mean epg for control (c) and treated (t) groups before (1) and after (2) treatment, respectively. The differential leukocyte count was analyzed based on the theory of generalized mixed linear models, using the Poisson distribution with the GLIMMIX procedure in the Statistical Analysis System program (SAS System, Inc., Cary, NC, USA). Tukey’s test was performed to determine significance.

## Results

**Figure 1:**
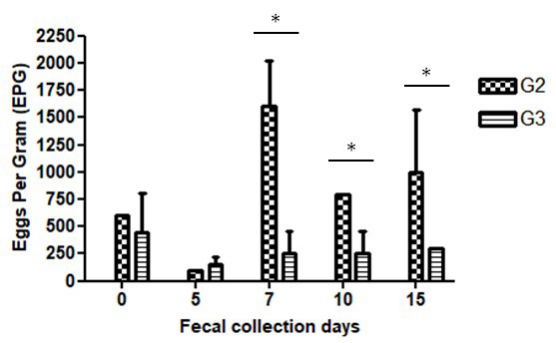
Mean number ± standard error of eggs per gram of feces (epg) observed in mice infected with *H. polygyrus bakeri* and treated with 3% DMSO (G2) and those treated with *C. citratus* aqueous extract (G3). Significant differences were revealed by the Tukey post hoc test after ANOVA (*p *<* 0.05).

**Figure 2:**
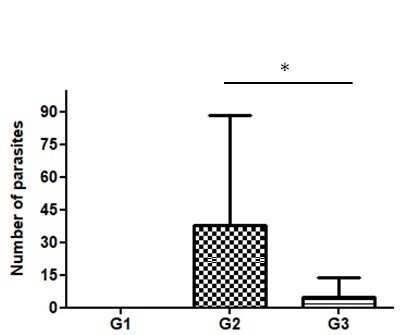
Mean number ± standard error of adult *H. polygyrus bakeri* found in noninfected mice (G1), in mice infected with *H. polygyrus* and treated with 3% DMSO (G2), and in mice treated with *C. citratus* aqueous extract (G3). Significant differences were revealed by Tukey post hoc test after ANOVA (*p *<* 0.05).

*Heligmosomoides polygyrus bakeri* nematode eggs were observed in the feces of infected mice (G2 and G3) 15 days after the administration of *C. citratus* crude aqueous extract (Figure 1). The epg remained similar between groups G2 and G3. On day seven and thereafter, G2 showed a significantly higher epg (p<0.05) indicating a reduction in *H. polygyrus bakeri* eggs (54%). The number of adult nematodes in the small intestine of infected animals showed an average of 43.4 parasites (G2), while the average in the infected group treated with *C. citratus* (G3) was 4.87 parasites (Figure 2), i.e., significantly different (p<0.05) indicating a reduction in *H. polygyrus bakeri* adults (89%).

The blood count did not vary significantly between groups. However, the differential leukocyte count (Table 1) revealed a difference in these cells between the infected groups. A comparison of G2 and G3 showed a difference between the levels of neutrophils (9.1-4.4%), monocytes (5.4-7.4%), lymphocytes (80.8-86.1%), and eosinophils (2.0-0.5%).

**Table 1:** Mean leukocyte counts ± standard error in mice of the C57BL/6 strain: noninfected (G1); infected with *H. polygyrus bakeri* and treated with 3% DMSO (G2); and infected with *H. polygyrus bakeri* and treated with *C. citratus* aqueous extract (G3).

Group	Leukocytes					
	**Neutrophils**	**Monocytes**	**Lymphocytes**	**Eosinophils**	**Basophils**	**Rods**
G1	4 ± 1.9^a^	5.8 ± 2.0^a^	90^a^	0^a^	0^a^	0.2^a^
G2	9.1 ± 3.6^b^	5.4 ± 3.3^a^	80.8 ± 4.0^b^	2.0 ± 1.0^b^	0.3 ± 0.3^a^	0.1 ± 0.2^a^
G3	4.4 ± 1.0^a^	7.4 ± 2.3^b^	86.1 ± 2.2^a^	0.5 ± 0.5^a^	0.4 ± 0.5^a^	0.2 ± 0.3^a^

Different letters in the same row indicate a statistical difference. P-value <0.0001.

**Figure 3:**
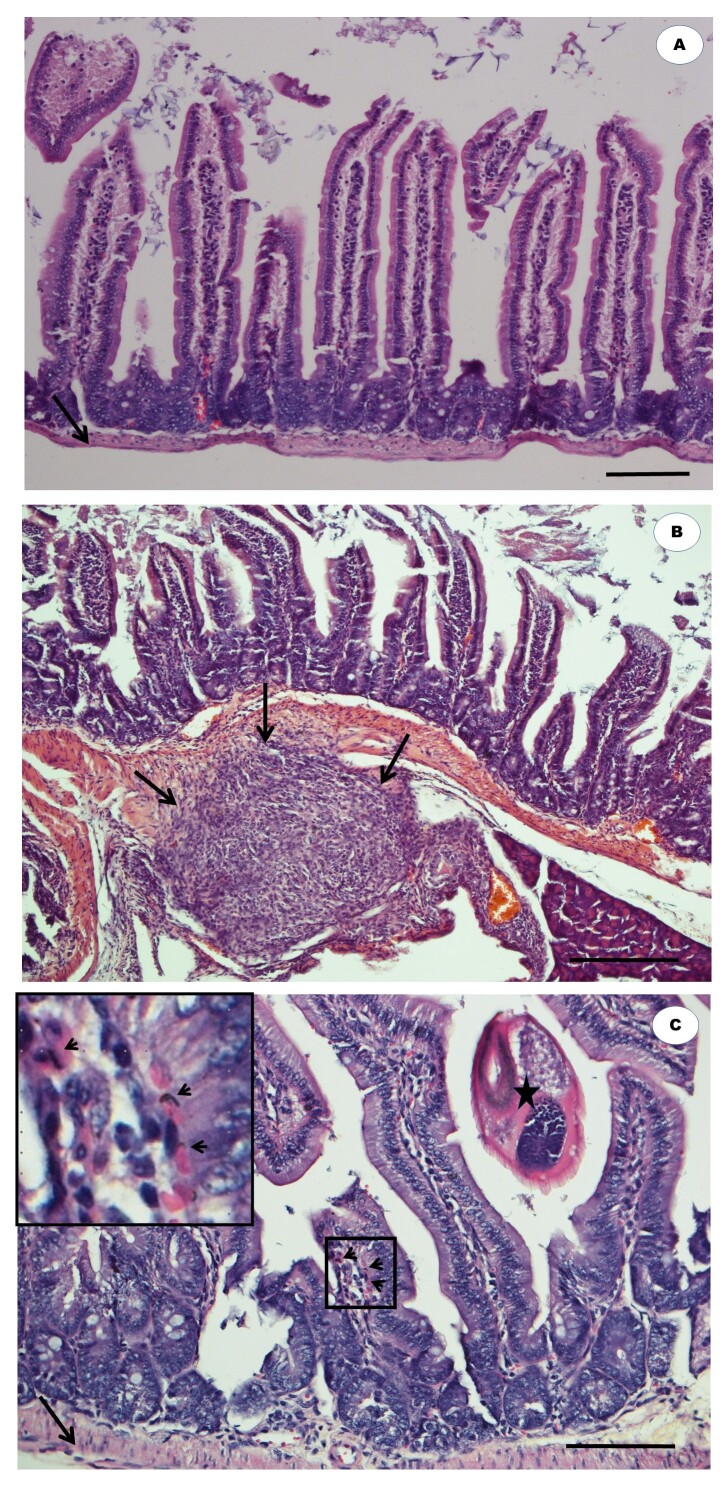
Photomicrograph of a section of the small intestines of mice infected and noninfected with *H. polygyrus bakeri* and treated or not with *C. citratus*. Noninfected control shows normality in its basal lamina (A). Negative control (3% DMSO) shows granulomatous enteritis (chronic active) in the muscle layer (B). The group treated with *C. citratus* showed mild enteritis with a few eosinophils in the lamina propria of the villi (C). Black arrows point to the basal lamina, black arrowheads point to the eosinophilic infiltrate (inset), and the star indicates the nematode. Bars: 100 µm.

The anatomo-histopathological analysis showed no lesions in the small intestine of the mice in the non-infected G1 (Figure 3A). On the other hand, signs of active chronic granulomatous enteritis were detected in the muscle layer of individuals in G2 (Figure 3B), and eosinophilic infiltrates were identified in the lesions. After treatment with *C. citratus*, the histology results of G3 were similar to those of G1 (Figure 3C), but with mild and moderate lesions. No differences were found in the anatomo-histopathology of the hepatic and renal parenchyma of the three groups.

## Discussion

Anthelmintic drugs are the principal way to control nematodes; however, anthelmintic resistance has led to the need for alternative methods of control. Medicinal plants have gained attention for containing secondary compounds with anthelmintic activity, which has been detected especially through *in vitro* tests. However, far fewer *in vivo* assays have been performed than *in vitro* tests, indicating the need for further studies in this area. Here, it was demonstrated that aqueous extract of *C. citratus* administered orally to mice infected with the nematode *H. polygyrus bakeri* has anthelmintic and possibly anti-inflammatory activity.

Potential anthelmintic drugs may undergo modifications that can negatively affect their efficacy when in contact with animal metabolisms. Thus, *in vivo* anthelmintic assays are necessary to prove the effects of a potential treatment molecule. The results obtained here indicate a reduction in the number of *H. polygyrus bakeri* eggs (54%) and adults (89%) in the group treated with *C. citratus* aqueous extract.

Studies with other plants have also shown efficacy when evaluated *in vivo*. Although Githiori et al. (2003) did not find a reduction in the number of nematode eggs in mice infected with *H. polygyrus bakeri*, they found that the administration of 500mg/kg of aqueous extract of *Albizia anthelmintica* bark reduced the number of adult parasites. After infecting mice with *H. polygyrus bakeri*, Wabo Poné et al. (2009) demonstrated the effectiveness of ethanolic extract of *Canthium mannii* at all the tested concentrations, except at the lowest dose of 350mg/kg. At a dose of 600mg/kg, they achieved a 75% efficacy rate in reducing the number of eggs and of 83.6% of adult worms. In both studies, the extract concentrations were higher than those used in the current study, suggesting that the aqueous extract obtained from *C. citratus* was more effective.

A few *in vivo* studies with *C. citratus* have analyzed its anthelmintic activity. Macedo et al. (2015) observed a 38.5% reduction in *H. contortus* in the gerbil *Meriones unguiculatus* after treatment with 800mg/kg of *C. citratus* essential oil. In another study, Macedo et al. (2019) used *C. citratus* essential oil at a concentration of 500mg/kg and its nanoemulsion at 450mg/kg in sheep and achieved a reduction of 66.4 and 83.1% of *H. contortus*, respectively. In contrast, Rodenbücher et al. (2023) found that treatment with 1g/bird/day of *C. citratus* essential oil had no effect against natural and experimental *Ascaridia galli* infection in laying hens. The results obtained with *C. citratus* aqueous extract showed greater efficacy in reducing the parasite load of nematodes in mice, since the maximum concentration used was 50mg/kg.

Changes in blood profile are common during nematode infection, such as a decrease in red blood cells and an increase in eosinophils and immunoglobulin E (IgE). In this study, leukocyte changes such as increased neutrophils and eosinophils were found during *H. polygyrus bakeri* infection. A reduction in neutrophils and eosinophils after treatment with *C. citratus* aqueous extract was also identified. Similarly, Monroy & Enriquez (1992) demonstrated the occurrence of several changes in tissue and blood, such as leukocytosis, neutrophilia, and eosinophilia, in murine mice infected with *H. polygyrus bakeri*. During the course of *H. polygyrus bakeri* infection there was an increase in interleukins involved in the inflammatory reaction and in eosinophilic infiltrate in the intestine of Balb/c mice (Doligalska et al., 2006). Eosinophils are cells of the immune system that are able to recognize helminth derivatives and modulate the inflammatory response in parasitized tissue (Shin et al., 2009). The anatomo-histopathological analysis in the present study revealed the presence of eosinophilic infiltrates in the muscle wall of the small intestine of infected mice. Based on these findings, the tests with mice indicate that treatment with *C. citratus* aqueous extract not only decreased the epg and the adult parasite load but possibly induced a process of lesion stabilization and reduction.

The presence of polyphenols, tannins, and flavonoids in *C. citratus* may contribute to its topical anti-inflammatory effect, making it suitable for the treatment of inflammatory skin conditions (Costa et al., 2016). Furthermore, the presence of phytosterols has shown anti-inflammatory activity. Hexane extract and stigmasterol from *Eryngium foetidum* leaves reduced auricular edema induced by 12-0-tetradecanoylphorbol acetate (TPA) in mice (García et al., 1999).

In another study, phytosterol compounds (ergosterol, β-sitosterol, stigmasterol, campesterol, and ergosterol acetate) were evaluated and found to reduce the inflammatory reaction in macrophage models induced by lipopolysaccharide (LPS). In addition, they inhibited cellular phagocytosis, nitric oxide (NO) production, tumor necrosis factor-α (TNF-α) release, and the expression and activity of the pro-inflammatory mediator cyclooxygenase-2 (COX-2), inducible nitric oxide synthase (iNOS), and phosphorylated extracellular signal-regulated protein kinase (p- ERK) (Yuan et al., 2019). Campesterol, stigmasterol, α-sitosterol, and β-sitosterol were some of the major components found in *C. citratus* methanolic extracts (Rocha et al., 2020). Therefore, it is possible that in addition to acting directly on the parasite and facilitating its mortality, *C. citratus* may also contribute to tissue recovery from injuries caused during infection.

## Conclusions

Based on *in vivo* anthelmintic assays, it was demonstrated that the aqueous extract obtained from *C. citratus* has high anthelmintic potential and does not represent risk during its administration, since the experimental mice showed no alterations at the analyzed concentration. Furthermore, an interesting reduction in intestinal lesions caused by *H. polygyrus bakeri* after treatment with *C. citratus*. These results suggest the promising potential for new research on the use of *C. citratus* as an alternative method to control gastrointestinal nematodes and possible concomitant wound healing activity of injured tissues.
